# Antiproliferative Activity of Prodigiosin Derived From *Serratia marcescens* VITSD2: An In Vitro and In Silico Approach

**DOI:** 10.1002/mbo3.70106

**Published:** 2025-12-03

**Authors:** Shah Alam, Suraj Kumar Nag, Jemima Naine S., Mohanapriya A., Sreelakshmi R. Nair, Tamil Bharathi Palanisamy, Mohanasrinivasan V., Subathra Devi C.

**Affiliations:** ^1^ School of Bio Sciences and Technology Vellore Institute of Technology Vellore Tamil Nadu India

**Keywords:** anticancer, antioxidant, Hep‐G2, pigments, prodigiosin, *Serratia marcescens* VITSD2

## Abstract

The red color pigment prodigiosin is a potent antioxidant produced by different strains of *Serratia marcescens* and other bacteria. The bio pigment demonstrates many hopeful impending bioactivities. Prodigiosin is an active proapoptotic agent against various cancer cell lines. In the present study, pigment produced from soil isolate *Serratia marcescens* VITSD2 was characterized and identified using UV, FTIR, GC‐MS and NMR analysis (^1^H NMR and ^13^C NMR). The antiproliferative activity of prodigiosin pigment from *Serratia marcescens* VITSD2 was evaluated on cancer cell lines. The active sites and binding patterns of molecular marker survivin was analyzed on docking against prodigiosin.A strong antioxidant potential was noticed at 5 mg/mL concentration with 70 ± 0.08% scavenging activity (2,2‐diphenyl‐1‐picrylhydrazyl)‐DPPH. The dose dependent inhibition of HepG2 cell proliferation was observed maximum with 67 ± 0.08% cytotoxic activity at 50 µg/mL. When compared to other cell lines, A549, HL 60 and MCF‐7, prodigiosin had a strong inhibitory activity on HepG2 cells. The Rf value of single band obtained in chromatography showed a value of 0.45. Maximum absorbance was observed at 535 nm. The pigment revealed the characteristic functional properties of the prodigiosin. On docking, the lowest binding energy exhibited was found to be ‐5.15 kcal/mol. The RMSD analysis indicated that the backbone structure converges at 18 ns before it attains stability. Pigment production from *Serratia marcescens* VIT SD2 offer a renewable and sustainable alternative to synthetic pigments, reducing dependence on nonrenewable resources. The study outcomes specified that the bio pigment prodigiosin extracted from *Serratia marcescens* VIT SD2 is a promising drug candidate for therapeutics.

## Graphical abstract

1. Prodigiosin pigment from *Serratia marcescens* VITSD2 was structurally characterized using UV, FTIR, GC‐MS, and NMR techniques.
2. It showed strong cytotoxic activity on HepG2 cells (67 ± 0.08% at 50 µg/mL), outperforming effects on A549, HL60, and MCF‐7 lines.
3. Molecular docking with survivin revealed stable binding ( − 5.15 kcal/mol), supporting its proapoptotic role.
4. The pigment exhibited 70 ± 0.08% antioxidant activity at 5 mg/mL, highlighting its therapeutic potential as a sustainable natural drug.

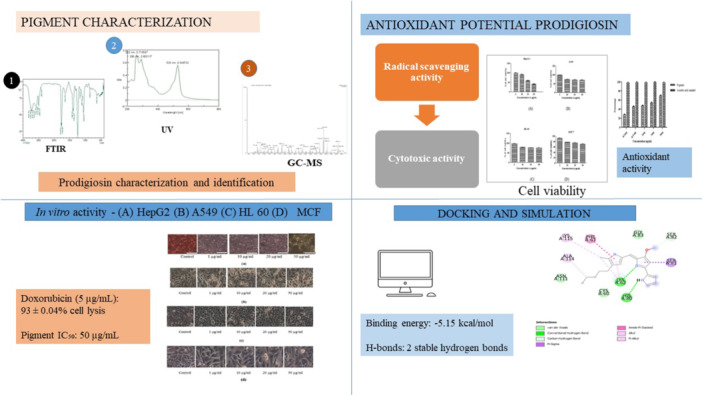

## 1 Introduction

One of the world's topmost illnesses is cancer. The major cause of death worldwide currently is cancer. According to Arbyn et al. (2020), the proportion of cancer patients is steadily rising and is the disease with the highest global death rate. Aside, from cost, other factors that influence the choice of anticancer medication throughout treatment include the drug's in vivo metabolic endurance, targeted action, immunological reactivity, and the likelihood and severity of adverse effects. There are currently very few effective chemotherapeutic drugs available to treat neoplasmic cancer. Current medications frequently have harmful side effects in noncancerous cells and normal tissues. The creation of novel drug formulations and the hunt for safe compounds are two approaches to address the critical issue of the safety of chemotherapy drugs. Cancer is among the most common diseases in the world. Cancer is the greatest cause of death worldwide. According to Johnstone et al. (2002), most chemotherapy drugs contain anticancer action that triggers apoptosis. Numerous natural and artificial product sources have the capacity to cause cancer cells to undergo apoptosis (Johnstone et al. 2002; Gordon et al. 2007; Gohil et al. 2021; Gunardi and Timotius 2023). One of the most important sources of chemical combinations with potential medical applications is natural goods. The secondary metabolism of organisms greatly benefits from the pigments produced by them. These days, microbiological sources provide anticancer medicines that are easily obtained and require little expense. More and more claims are being made about microbial metabolites being less costly, safer, and more effective. In cancer therapy, numerous medications are being tested in clinical settings. Notwithstanding their clinical utility, these medications come with a plethora of side effects and are somewhat costly. To make anticancer agents more cost‐effective and better designed, additional research will be done. As a result, the research for novel anticancer drugs is imperative, and there is more opportunity to find new agents. One of the key molecules that significantly contributes to bioactivity is prodigiosin. Prodigiosin's cytotoxicity and co‐nuclease activity are both significantly influenced by the A‐pyrrole ring (Montaner and Pérez‐Tomás 2003; Pérez‐Tomás et al. 2003). *Serratia marcescens* produces prodigiosin, which has a distinct chemical structure that could change how new anticancer medications are made. Prodigiosin is an indication of exceptional anticancer action. Prodigiosin could cause several malignant cell types to undergo apoptosis while having little negative effects on healthy cells (Anwar et al. 2020; da Silva Melo et al. 2000). This compound acts through several different ways. Prodigiosin affects several essential cellular processes that play a major role in its anticancer activity. Prodigiosin's pharmacology is known to have bioactive qualities, and because of its possible anticancer effects, researchers have recently expressed interest in the drug (Nguyen et al. 2022). The main objective of the study was to characterize the pigment extracted from *Serratia marcescens* VITSD2 and evaluate the anticancer activity through in vitro analytical assays and in silico docking analysis. Hence the main scope of this study is to explore prodigiosin pigment as a potential compound for cancer therapy which is both effective and safe, with remarkable uniqueness. Prodigiosin pigment offers new hope for cancer patients. This study advances beyond previous research works by evaluating anticancer activity in a single comprehensive analysis of an environmentally isolated strain, which could be a promising source of prodigiosin for anticancer activity. In addition to it *S. marcescens* is the natural producer of has vigorous growth characteristics, environmental adaptability, and metabolic versatility made the strain a promising producer of prodigiosin.

## 2 Materials and Methods

### 2.1 Experimental

The pigment was extracted from *Serratia marcescens* VITSD2 isolated from soil (Gen Bank accession number: HQ197382). The bacterial culture of *Serratia marcescens* VITSD2 was used in this study for further pigment analysis. For both the wild type and the mutant strain, 10 mL of the inoculum was added to 90 mL of sterile production broth. The mixture was then shaken at 27°C for 24 h, and the crude was centrifuged at 10,000 rpm Choosing an appropriate solvent to extract the bacterial pigment was the first step in the pigment extraction process. Ethanol, acetone, methanol, petroleum ether, ethyl acetate, chloroform, hexane, diethyl ether, and distilled water were among the different solvents utilized (Devi et al. 2013).

### 2.2 Characterization of the Pigment Produced From *Serratia marcesens* VIT SD2

Thin‐layer chromatography was also used, along with an analysis of the purified pigment's absorbance. The UV spectrophotometer was used to investigate the pigment's color intensity. By measuring the absorbance spectrophotometrically, prodigiosin quantification was expressed per cell (A535 mL^−1^ OD600) using the relative prodigiosin concentration. In 2008, Boussaada et al. To verify the prodigiosin's functional groups and signature areas, the purified enzyme was subjected to GC‐MS analysis and Fourier‐Transform Infrared Spectroscopy. Prodigiosin's NMR data was also acquired to validate the pigment's structure. According to Boussaada et al. (2008), the pigment was extracted from *Serratia* using the following solvents: petroleum ether, acetone, diethyl ether, methanol, ethyl acetate, hexane, ethanol, distilled water, and chloroform. The extracted pigment was further analyzed for FTIR, GC‐MS and NMR analysis.

### 2.3 Fourier‐Transform Infrared Spectroscopy and GC‐MS Analysis

Using a Thermo Nicolet Avatar 370 spectrometer, the pure pigment sample was examined by FT‐IR spectroscopy (Freshney et al. 1982). To connect the functional groups in the pigment with prodigiosin and its derivatives, GC‐MS analysis was performed on the pigment. After that, a comparison was made using the earlier reports (Boussaada et al. 2008).

### 2.4 NMR Analysis

**“**The structure of Prodigiosin was confirmed by NMR (^1^H‐NMR and ^13^C‐NMR).^1^H‐NMR spectra was recorded on a Bruker AM 300 MHz spectrometer (Spectrospin, Fallenden, Switzerland)” (Mosmann 1983) (Gerber 1975).

### 2.5 Determination of Antioxidant Activity

**“**The antioxidant activity was determined by DPPH scavenging assay at various concentrations (0.1, 0.5, 1.0, 3.0 and 5.0 mg/mL)” (Boger and Patel 1988) (Williams et al. 1971). Ascorbic acid was used as reference (5.0, 3.0, 1.0, 0.5, and 0.1 mg/mL). The percentage of radical scavenging activity was measured.

### 2.6 Cell Viability Assay

The National Centre for Cell Science (NCSS) in Pune provided the cell lines (HepG2, HL 10, MCF7, A549), which were then cultivated in RPMI‐1640 media (Carbonell et al. 1996, 2000). To evaluate the cell viability, the MTT test was used (Budzikiewicz et al. 1964a; Hu et al. 2015). Each of the 5 × 10^3^ cell lines were planted into a well of 96 well plates. To evaluate the potential, 100 µL of the pigment in triplicate at different concentrations (1, 10, 20, and 50 µg/mL) was utilized. A 5 µg/mL dose of doxorubicin served as the internal positive control. At 560 nm, the quantity of formazan was measured with a microplate reader.

### 2.7 Evaluating the Therapeutic Scope of Prodigiosin With In Silico Analysis

The factors that contributed to the proliferation of hepatocellular carcinoma (HCC) cells were inhibition of apoptosis and activation of oncogenes which lead to blockage of signaling cascades mainly caspase‐9, caspase‐7 and caspase‐3 (Niraimathi et al. 2013). The antiapoptotic protein suppression by wild type p53 was studied and observed the overexpression of 70% of HCC from Asia. Hence survivin protein is a molecular biomarker of HCC.

### 2.8 Target Structure Retrieval

The crystalline structure of human Survivin was retrieved from Protein Data Bank, PDB (www.rcsb.org, PDB ID: 3UECA) (Guex et al. 2009) The structure was solved using x‐ray diffraction at a resolution of 2.18 Å. The residues were then energy minimized using the Swiss‐PdbViewer (Morris et al. 2009). The ligand prodigiosin was obtained from PubChem database and further energy minimized using CORINA Classic.

### 2.9 Molecular Docking

The interaction of protein with the ligand can be best determined using the docking studies. Here the AutoDock 4.2 software that has the basic principle of Lamarckian genetic algorithm was carried out on the active site of the protein surviving. (Binkowski 2003) Before docking, the protein structure was energy minimized, and all water molecules were removed. The binding pockets of the protein as well as the active site residues of the protein was documented using castP (Slater et al. 2003).

### 2.10 Molecular Dynamics Simulation Studies

Using GROMACS 4.5.5 (Groningen Machine for Chemical Simulations), the protein and ligand docked complex that was produced by the Autodock tool was put through MD simulation (Pronk 2013). The PRODRG server was used to generate the ligand coordinates, and the GROMOS96 43a1 force field was used to prepare the protein topology file. At 300 K and 1 bar of pressure, the system was energetically reduced and exposed to 20 ns MDS. The radius of gyration, RMSD, and RMSF were among the parameters that were evaluated.

## 3 Results

### 3.1 Characterization and Identification of the Pigment

Among the solvents such as petroleum ether, acetone, diethyl ether, methanol, ethyl acetate, hexane, ethanol, distilled water, and chloroform, ethyl acetate was selected as it showed the highest extraction efficiency and better solubility for the pigment.

### 3.2 Fourier‐Transform Infrared Spectroscopy and GC‐MS Analysis

**Figure 1:**
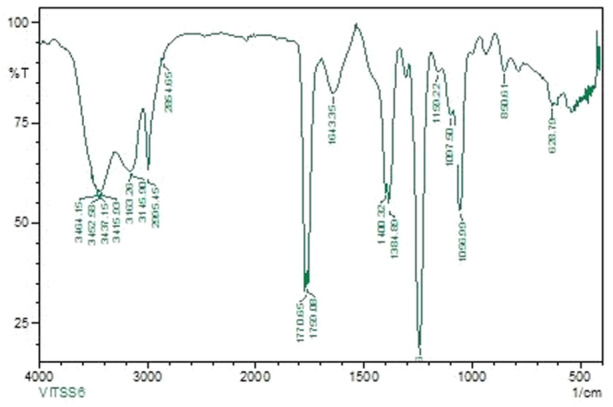
FTIR spectrum of prodigiosin from *Serratia marcescens* VITSD2.

**Figure 2:**
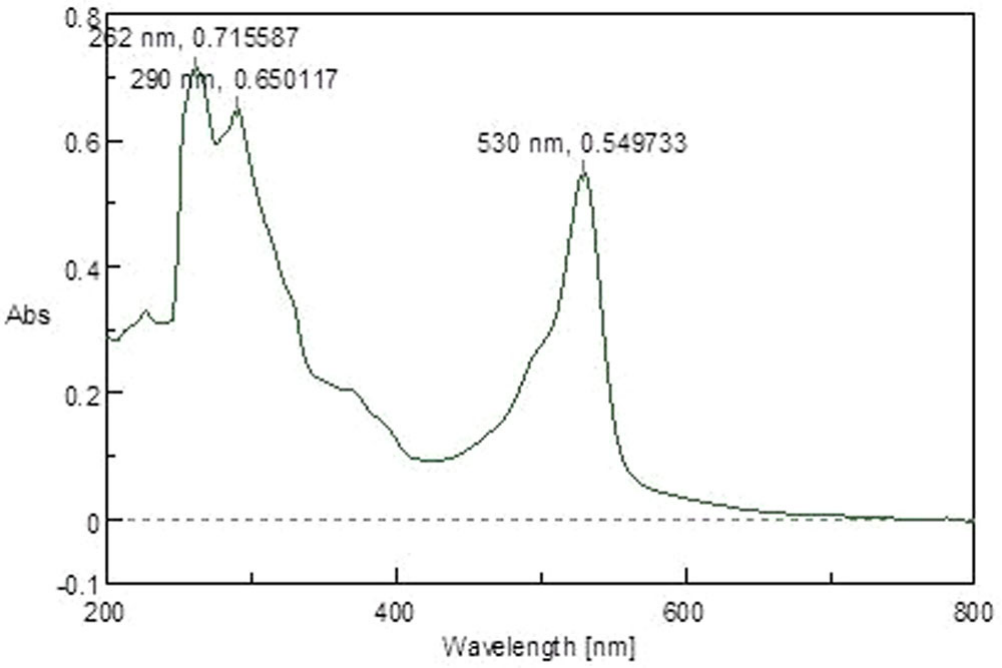
UV spectrum of prodigiosin from *Serratia marcescens* VITSD2.

**Figure 3:**
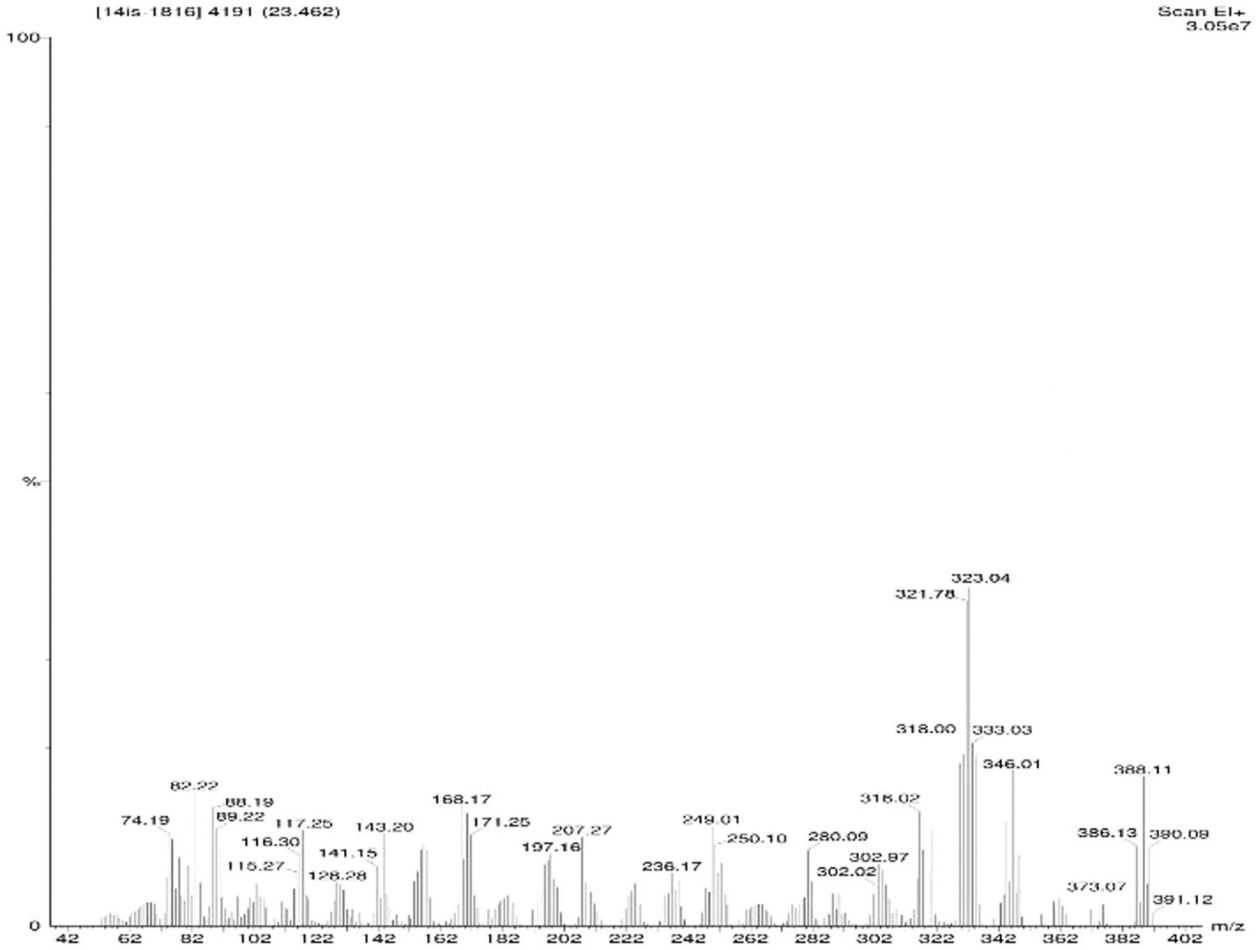
GC‐MS analysis of purified prodigiosin pigment produced from *Serratia marcesens* VITSD2.

FT‐IR absorption in KBr for the red pigment was dominated by strong bands at 2924.78 cm^‐1^ and 2853.67 cm^‐1^ (aromatic CH). This indicates that the pigment pattern is related to that of prodigiosin (Figure 1). During the UV‐Vis absorption spectra analysis the baseline for the spectrum observation was chosen between 300 and 750 nm. The maximum absorbance of pigment was obtained at 535 nm (Figure 2). The pigment produced from *Serratia marcescens* corresponds to 323.04 D *m*/*z*, thus confirmed the presence of prodigiosin. (Figure 3).

### 3.3 NMR Analysis

**Figure 4:**
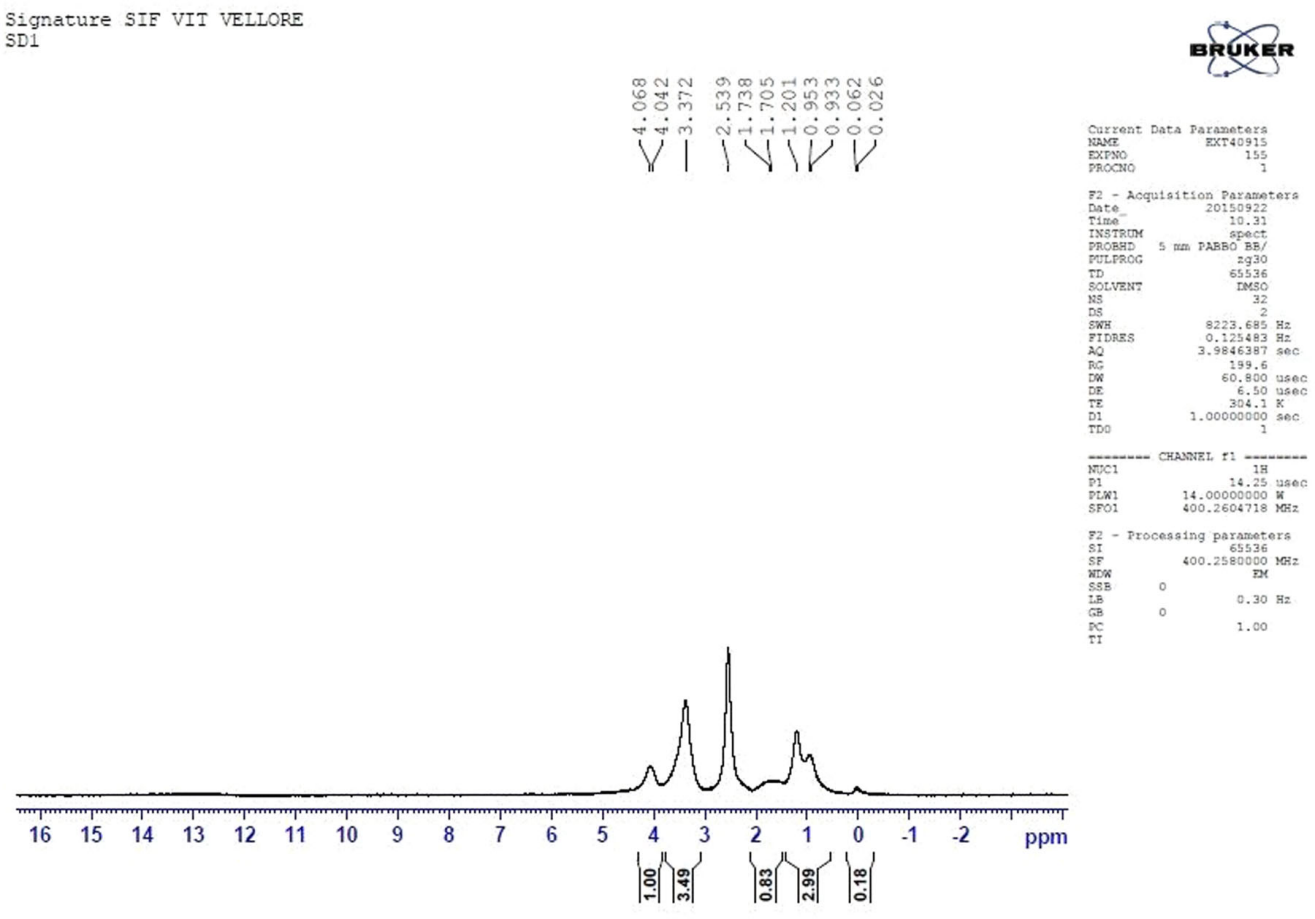
^1^H‐NMR spectrum of purified prodiogiosin UV spectrum of prodigiosin from *Serratia marcescens* VITSD2.

**Figure 5:**
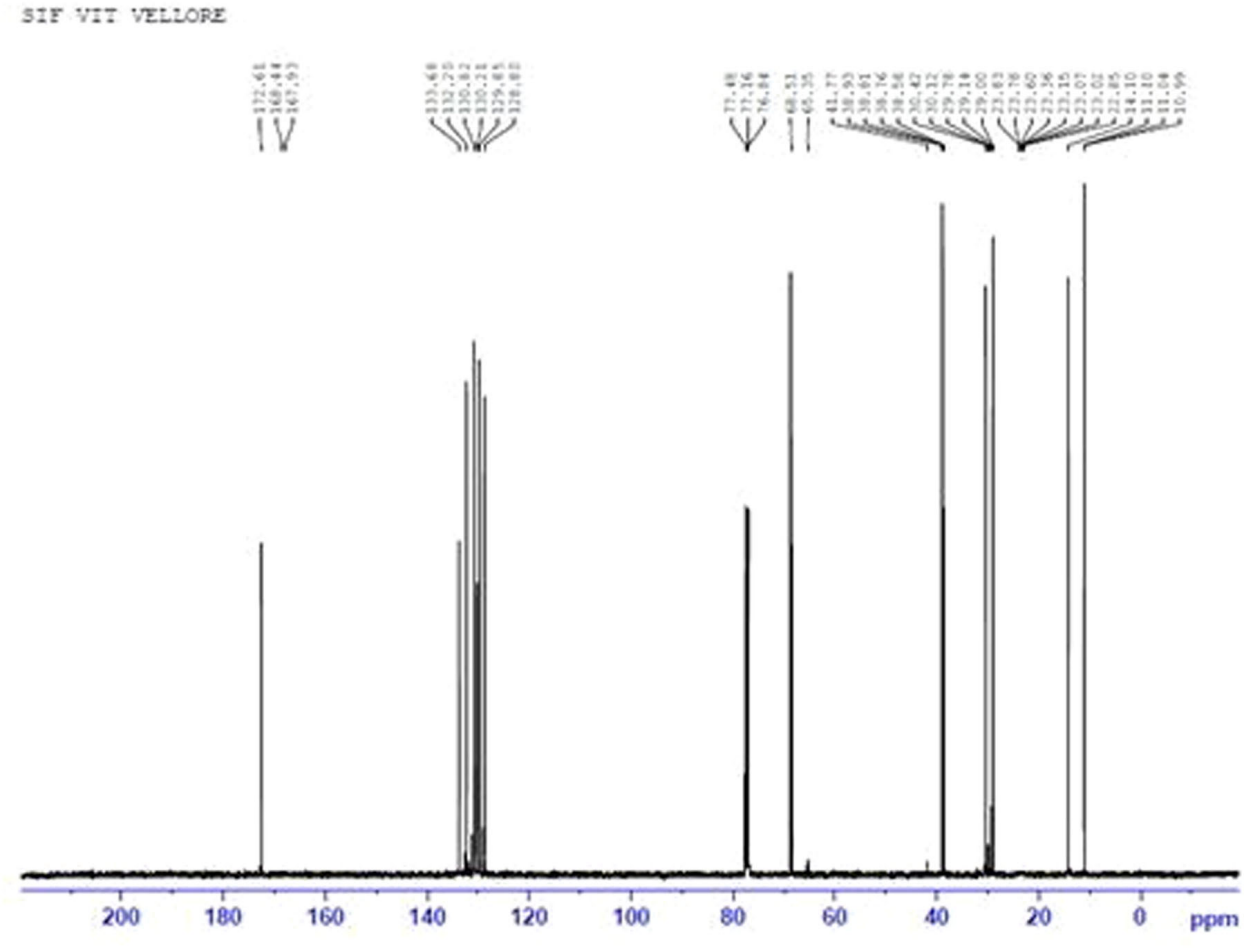
^13^C NMR spectrum of purified prodigiosin.

**Figure 6:**
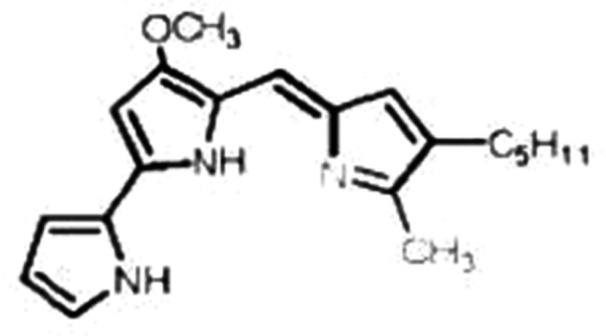
Chemical structure of prodigiosin.

The structure of prodigiosin was further confirmed by high‐field ^1^H‐NMR and ^13^C NMR spectroscopy. The mass spectrum, as illustrated in Figures 4 and 5 supports the structure of prodigiosin. ^1^H‐NMR (CDCl3) spectrum of prodigiosin represented the peaks corresponding to chemical shifts at 2.55 (3H, s), δ4.01 (3H, s), 2.39 (2H, m), 1.31 (4H, m), 1.54 (2H, m), 0.87 (3H, m) (Figure 6). ^13^C NMR at (CDCl3), 117.00 (ring B‐C3), δ120.7 (ring A‐C2), 92.81 (ring A‐C4), 126.95 (ring A‐C5), 165.79 (ring B‐C23), 122.27 (ring B‐C2), 92.81 (ring B‐C4), 58.69 (OCH3), 128.41 (ring C‐C4), 147.72 (ring B‐C5), 126.95 (ring C‐C5), 25.32 (C10), 29.69 (C20), 31.41 (C30), 14.01 (C50) (Figure 6).

### 3.4 Radical Scavenging Activity

**Figure 7:**
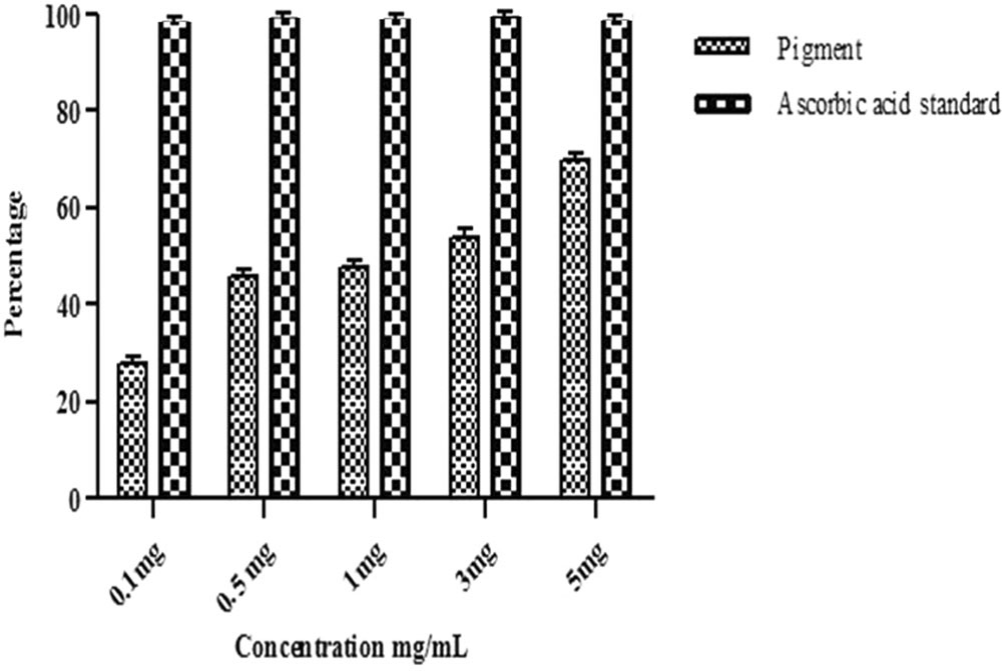
Antioxidant activity of pigment produced from *Serratia marcescens* VITSD2.

The purified prodigiosin exhibited antioxidant potential at a concentration of 5 mg/mL against DPPH was found to be exhibit antioxidant potential at 5 mg/mL with 70 ± 0.08% inhibition (Figure 7). The antioxidant activity was found highest at 10 mg/mL with 90 ± 0.08% inhibition.

### 3.5 Cytotoxic Activity

**Figure 8:**
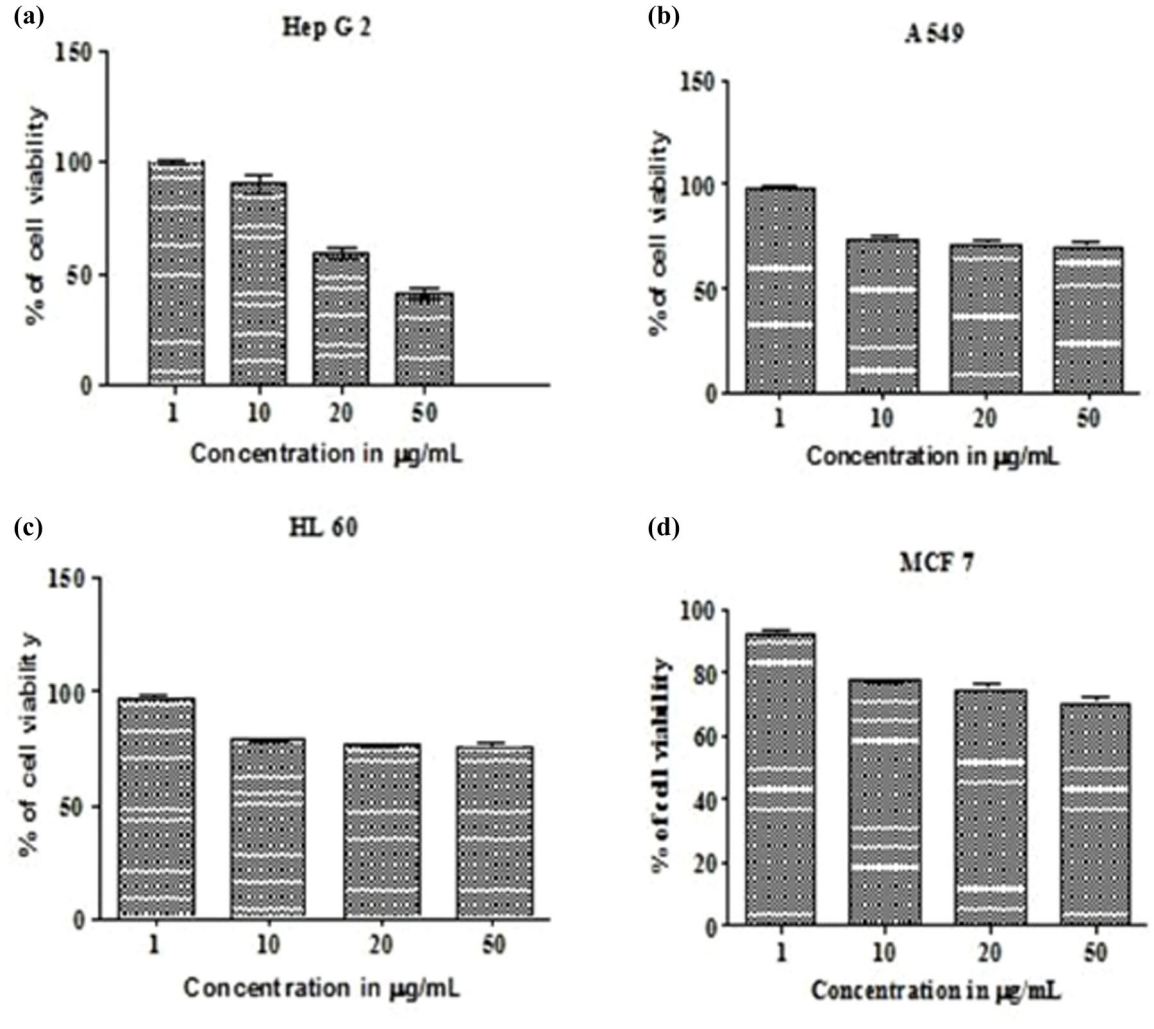
Percentage of cell viability shows the effective drug concentration toxic to cell lines (a) HepG2 (b) A549 (c) HL 60 (d) MCF7.

**Figure 9:**
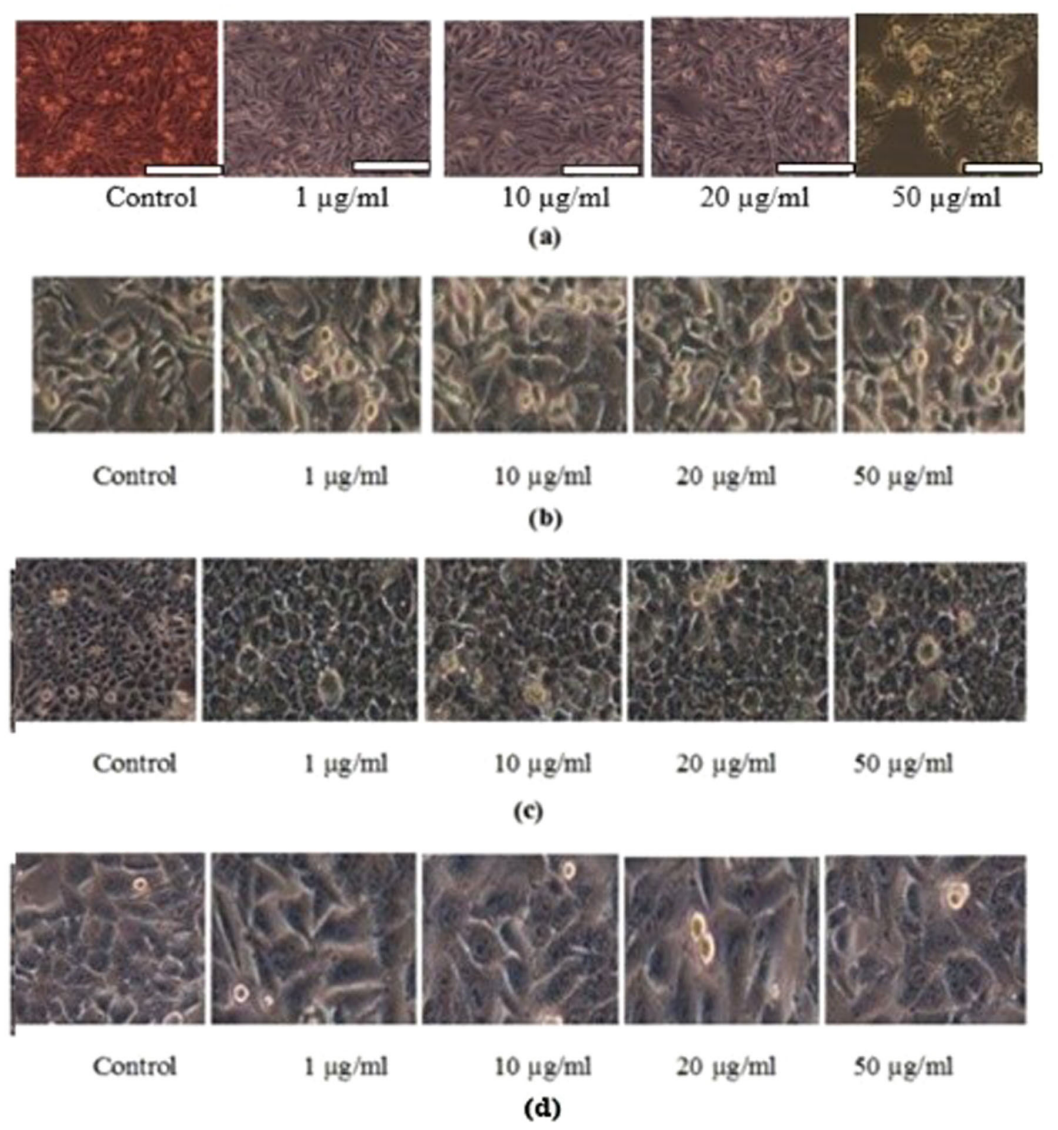
Morphological changes of cell lines treated with *Serratia marcescens* VITSD2 pigment prodigiosin (a) HepG2 (b) A549 (c) HL 60 (d) MCF 7.

The pigment prodigiosin was found to induce apoptosis in HepG2 cell lines by dose dependent manner. The HepG2 cell lines were observed with decrease in proliferation of cells at the IC50 of 50 µg/mL indicated a potent anticancer activity against HepG2 cells. The assessment of the cell viability revealed 43 ± 0.08% viability at 50 µg/mL concentration with 67 ± 0.08% cytotoxicity. On A549 cell lines at IC50 of 50 µg/mL, showed 70 ± 0.08% cell viability, on Human promyelocytic leukemia cells (HL 60) cell lines at IC_50_ of 50 µg/mL showed 75 ± 0.09% viability. The pigment tested in dose‐dependent effect on Michigan Cancer Foundation‐7(MCF 7) cell lines were noticed with cell death with IC50 of 50 µg/mL with 75 ± 0.07% viability. The positive control doxorubicin (5 µg/mL) which was used as internal positive control showed 93 ± 0.04% cell lysis (Figures 8, 9). The pigment also exhibited cytotoxicity against HepG2 cells in dose‐dependent manner with the IC50 value of 50 μg/mL with cytotoxicity of 67 ± 0.08% and 43% of cells were found to be viable.

### 3.6 Molecular Docking and Molecular Dynamic Simulation

#### 3.6.1 Molecular Docking

**Figure 10:**
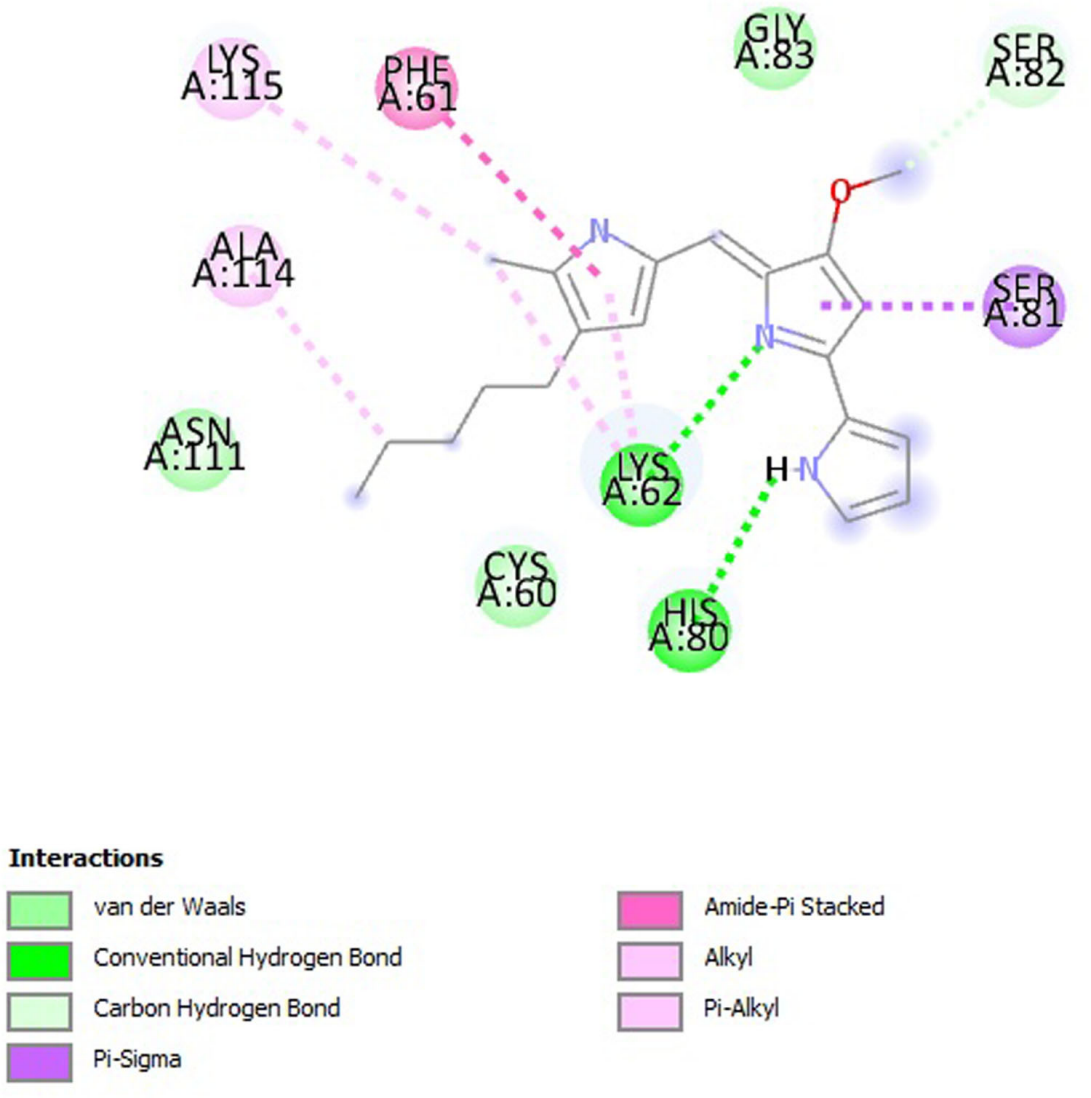
Molecular interaction between survivin and prodigiosin.

To ensure the robustness and reproducibility of the docking results, molecular docking was performed in three separate batches using the AutoDock software. A uniform grid size of 50 × 48 x 62 in diameter was used, centered at the coordinates (X = ‐26.776, Y = ‐8.801, Z = ‐6.302) of the target protein site. Among the three independent docking experiments, the second trial yielded the most favorable binding state, with a binding energy of ‐5.15 kcal per mole and two stable hydrogen bonds. This conformation further selected for molecular dynamics simulation (MDS). A corresponding 2D interaction diagram illustrating key binding interactions (Figure 10).

**Figure 11:**
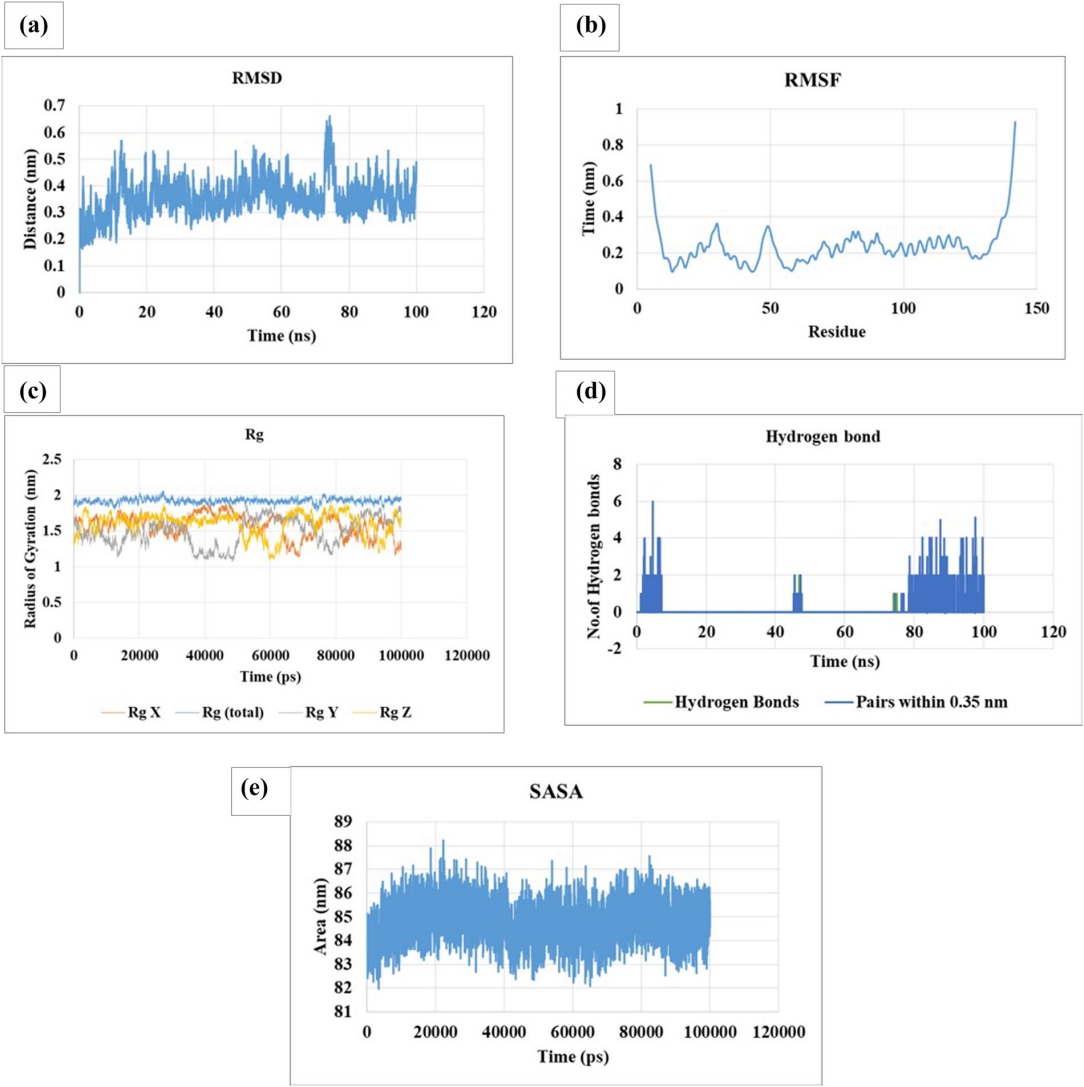
Molecular dynamics simulation analysis of the protein structure. (A) RMSD plot indicates overall structural stability. (B) RMSF highlights residue‐level flexibility, (C) Radius of gyration (Rg) shows consistent protein compactness, (D) hydrogen bond analysis displays transient interaction patterns and (E) SASA plot reveals stable solvent exposure with minor fluctuations throughout the simulation.

Root means square deviation (RMSD) analysis showed that the initial structural deviation increased rapidly in the first 10 ns and stabilized at a range of 0.3–0.4 nm. Throughout its 100‐ns trajectory, the system exhibited slight fluctuations, indicating no significant conformal changes. Occasional peaks, especially around ~75 ns and up to ~0.6 nm, indicated transient flexibility or local rearrangements of the structure. Despite these fluctuations, the RMSD has returned to a base level, reflecting structural resilience. The absence of a consistent upward trend suggested that the protein retained its universal fold. Overall, the system shows dynamic but stable behavior during the simulation period (Figure 11). The Root means square fluctuation (RMSF) plot showed that most residues had limited fluctuation and that the RMSF values ranged from 0.1 to 0.4 nm, indicating overall structural stability. However, significantly higher variations were observed in the N‐terminal region (residues ~1‐5) and in the C‐terminal region (residues ~145–150) with a peak of approximately 1 nm. These higher volatility rates indicated greater flexibility or disorder in the terminal areas, which are usually exposed to solvent and less structured. Small local peaks are observed at residues 40 and 80, indicating possible loop or flexible surface areas exposed to the solvent. The protein core (columns 20–120) showed relatively stable volatility. This structure is consistent with a protein that maintained its structural integrity while allowing flexibility in the final or loop regions.

The rotation profile (Rg) was analyzed over a simulation time scale of 100,000 ps to assess the structural compactness and stability of the molecule. The total Rg remained relatively stable around ~1.9–2.0 nm over the entire run, indicating overall conformal integrity without significant deformation. However, the Rg components along the X, Y and Z axes showed significant variations, especially on the Y axis, which decreased to a value of ~1.1 nm indicating an axis‐specific flexibility. Variability of RgY and RgZ indicated a dynamic conformal rearrangement, possibly caused by domain displacement or partial relaxation of the structure in the localized regions. Despite this, the overall Rg was not affected, which reinforces the fact that the fundamental structure remains compact. Consistency in Rg(total) together with directional flexibility indicate a structurally stable system.

Hydrogen bond analysis showed that during the initial 0–10 ns, there were transient interactions, with up to 6 H‐ bonds forming. The prolonged phase from 10 to 70 ns showed minimal or no formation of H‐ bonds, indicating an unstable or weak bond formation. Since approximately 75 ns, a steady increase in formation of hydrogen bonds has been observed. Peak values of 6‐7 hydrogen bonds indicate more stable interactions in the later simulation phase. The trend of atom pairs at 0.35 nm are very close to actual hydrogen bonds, which confirms meaningful interactions. Overall, the system shows dynamic fluctuations with eventual stabilization of hydrogen bonding.

The plot of the solvent accessible surface area (SASA) showed that the protein maintained a relatively stable exposure to the solvent during the 100‐ns simulation, fluctuating between approximately 82 and 87 nm. Initial increases in SASA during the first 20 ns indicated minor structural modifications or surface residue thickening. After this period, SASA values stabilized with a steady oscillation, indicating dynamic equilibrium with little conformal breathing. The absence of sharp drops suggested that there was no great collapse or disintegration. Mild fluctuations were expected due to transient chain movements and solvent interactions. Overall, the consistent SASA values indicate well maintained structure.

## 4 Discussion

The pigment produced from *Serratia marcescens* corresponds to 323.04 D *m*/*z*, thus confirmed the presence of prodigiosin (Figure 3). The tripyrrole‐shaped pigment known as prodigiosin is derived from microbial sources and has several biological characteristics (Sundaramoorthy and associates 2009; Niraimathi and colleagues 2013). Prodigiosin is a highly significant pigment due to its ability to inhibit the growth of cancer cells while causing minimal harm to healthy tissues. This selective cytotoxicity made PG a potential candidate for developing anticancer drugs. (Guryanov et al. 2020). Prodigiosin and its near derivative are known to generate a distinctive absorption at 534 nm on spectrometric examination (Budzikiewicz et al. 1964b) (Ahmad et al. 2012). Since the eluded fraction yielded a single red colored band, ethyl acetate was determined to be the best solvent for pigment extraction (Marchal et al. 2014). After thin‐layer chromatography using a solvent system of chloroform:methanol (95:5 v/v), an Rf value of 0.43 was found. The wavelength at which the highest absorption was observed was 535 nm. Strong bands at 2924.78 cm^‐1^ and 2853.67 cm^‐1^ (aromatic CH) were seen in the FT‐IR absorption for the red pigment in KBr. This indicated that the pigment pattern is related to prodigiosin (Figure 1). During the UV‐Vis absorption spectra analysis the baseline for the spectrum observation was chosen between 300 and 750 nm (Figure 2). IR patterns on FTIR were like the earlier reports where the band at 1631 cm^‐1^ which corresponds to C‐N and C‐C stretching. The band at 1450 cm^‐1^ was identical for N‐H stretch vibration present in the amide linkages of the proteins (Samrot et al. 2011) (Kim et al. 2008) The maximum absorbance of red pigment was compared with previous reports. This was confirmed by comparing the results of study carried out by Ahmad et al. 2012; Valente et al. 2008 and Monreal and Reese (1969). Prodigiosin's maximum absorbance spectrum was reported by Prakash et al. (2013), Valente et al. (2008), and Monreal and Reese (19691) at 534.6 and 535 nm. The pigment extracted from *Serratia marcescens* VITSD2 was found to have a maximum absorbtion range that was identical. The refined pigment's molecular weight of 323.04 Da was discovered; this value is consistent with earlier results (Binkowski et al. 2008; Sundaramoorthy,^2009^; Niraimathi et al. 2013). Purified prodigiosin's chemical changes were ascribed in line with previously published research (Marchal et al. 2014; Montaner et al. 2000; Siva et al. 2012). At 5 mg/mL, the isolated prodigiosin showed antioxidant potential with 70 ± 0.08% inhibition (Figure 7). It was discovered that the pigment prodigiosin caused dose‐dependent apoptosis in HepG2 cell lines. The HepG2 cell lines were observed with decrease in proliferation of cells at the IC50 of 50 µg/mL. This indicated a potent anticancer activity against HepG2 cells. The assessment of the cell viability revealed 43 ± 0.08% viability, at 50 µg/mL concentration with 67 ± 0.08% cytotoxicity. A549 cell lines demonstrated 70 ± 0.08% cell viability at an IC50 of 50 µg/mL, while human promyelocytic leukemia cells (HL 60) demonstrated 75 ± 0.09% vitality at the same IC50. On Michigan Cancer Foundation‐7 (MCF 7) cell lines, the pigment was tested in a dose‐dependent manner. Cell death was observed with an IC50 of 50 µg/mL and 75 ± 0.07% viability. Figures 8 and 9 reveal that the internal positive control, doxorubicin (5 µg/mL), demonstrated 93 ± 0.04% cell lysis. The pigment was discovered to have an IC50 value of 50 μg/mL and shown considerable cytotoxicity against HepG2.

To ensure the robustness and reproducibility of docking results, molecular docking was performed in three independent batches using AutoDock software. A consistent grid size of 50 × 48 × 62 Å was employed, centered at the coordinates (X = ‐26.776, Y = ‐8.801, Z = ‐6.302), targeting the active site of the survivin protein. Among the three docking trials, the second batch yielded the most favorable binding conformation, exhibiting a binding energy of ‐5.15 kcal/mol and forming two stable hydrogen bonds. Further interaction analysis revealed that the ligand prodigiosin formed two π–σ interactions with residues HIS77 and SER88, along with one alkyl interaction with LYS78, indicating a stable association within the active pocket. The 2D interaction map of the docked complex is visualized using Discovery Studio (Figure 10). To assess the structural stability and dynamic behavior of the prodigiosin survivin complex, MDS was carried out using GROMACS 4.5.5 with the GROMOS96 43a1 force field. A 100 ns simulation was performed to evaluate the backbone stability, flexibility, compactness, hydrogen bonding, and solvent accessibility of the complex. The root mean square deviation (RMSD) trajectory revealed that the complex exhibited an initial increase in deviation during the first 10 ns, which then stabilized within a range of 0.3–0.4 nm. Throughout the 100‐ns trajectory, only slight fluctuations were observed, with transient peaks—particularly around ~75 ns reaching ~0.6 nm—indicating localized flexibility or minor structural rearrangements. Importantly, the RMSD values consistently returned to baseline levels, suggesting resilience of the protein–ligand complex and maintenance of its universal fold. These observations collectively point to a dynamically stable system with no significant conformational drift (Figure 11A). The root mean square fluctuation (RMSF) profile showed that most residues experienced minimal fluctuation (0.1–0.4 nm), confirming structural stability. However, notable peaks were observed in the N‐terminal ( ~ residues 1–5) and C‐terminal ( ~ residues 145–150) regions, where fluctuations reached approximately 1.0 nm. These fluctuations likely correspond to solvent‐exposed and less structured terminal regions. Minor peaks at residues 40 and 80 further suggest flexible loop regions. In contrast, the core region (residues 20–120) showed low volatility, consistent with a compact and stable protein core (Figure 11B). The radius of gyration remained stable throughout the simulation, fluctuating modestly between ~1.9 and 2.0 nm, which indicates conformational integrity and no major structural collapse. When decomposed by axis, significant variation was observed in Rg along the Y‐axis, which dropped to ~1.1 nm, indicating axis‐specific flexibility. These fluctuations may reflect partial relaxation or dynamic rearrangement of certain structural domains. Nonetheless, the stability of the total Rg values confirms the compact nature of the protein–ligand complex (Figure 11C). Hydrogen bonding between the ligand and protein was transient during the first 10 ns, with up to 6 hydrogen bonds forming sporadically. From 10 to 70 ns, minimal hydrogen bond formation was observed, suggesting a weaker or less stable interaction during this phase. Notably, after 75 ns, the number of hydrogen bonds gradually increased, reaching stable values between 6 and 7, indicating enhanced binding stability in the later stages of simulation. Atom‐pair distances consistently around 0.35 nm further validate the presence of meaningful hydrogen bonding interactions (Figure 11D). SASA analysis demonstrated a relatively consistent surface exposure to the solvent, with values ranging between 82 and 87 nm² throughout the 100‐ns trajectory. An initial increase during the first 20 ns indicated minor structural adjustments or expansion of surface‐exposed residues. Subsequently, the SASA values stabilized and oscillated moderately, reflecting a dynamic but equilibrium state. No sudden drops were observed, ruling out any major structural collapse or compaction events. The stable SASA values further reinforce the structural integrity of the complex during simulation (Figure 11E). Collectively, these molecular dynamics findings underscore the structural resilience and binding stability of the prodigiosin–survivin complex. Survivin, being both a genetic marker and a therapeutic target in HCC, has been associated with aberrant activation of the Wnt/β‐catenin pathway and inhibition of apoptotic processes (Yenkejeh et al. 2017). Prodigiosin's ability to form stable interactions with survivin suggests its potential role in restoring apoptotic activity and inhibiting cancer cell proliferation in HepG2 cells. Survivin is overexpressed in approximately 70% of HCC cases in Asia and is typically suppressed by wild‐type p53. Its abnormal overexpression often correlates with nuclear p53 positivity and mitotic stability, thus promoting cancer progression (Kannangai et al. 2005; Giodini et al. 2002). Targeting survivin represents a promising therapeutic strategy, and the current computational analysis supports the potential of prodigiosin as a survivin inhibitor. Previous studies have demonstrated prodigiosin's anticancer activity in various cancer cell lines, including A549, HepG2, MCF‐7, and WiDr (Islan et al. 2022), primarily through ATP depletion, mitochondrial dysfunction, and DNA damage (Tomás et al. 2001; Islan et al. 2022). Additionally, prodigiosin inhibits cancer cell migration and invasion by targeting key signaling pathways such as Wnt/β‐catenin. It has shown efficacy in both hormone‐sensitive and drug‐resistant breast cancer cell lines (Elahian et al. 2013; Wang et al. 2016). Prodigiosin derived from *Serratia marcescens* QBN VTCC 910026 demonstrated strong cytotoxic effects against MCF‐7, KB, and LU‐1 cell lines (Nguyen et al. 2022), while another strain, EMS 5, showed weaker effects against HepG2 cells. In the present study, prodigiosin isolated from *S. marcescens* VIT SD2 exhibited potent cytotoxic activity against HepG2 cells and showed promising results across other cancer cell lines under laboratory evaluation. These findings highlight the therapeutic potential of prodigiosin from *S. marcescens* VIT SD2, especially as an anticancer agent targeting survivin in HCC.

## 5 Conclusion

The interest in prodigiosin production is increasing because of the biological importance. Hence the industrial importance of this pigment also rises on large scale. Getting prodigiosin pigment from *Serratia marcescens* VITSD2 for medicinal uses is the main goal of this effort. Consumers are now using less expensive, safer pigments with improved potency and applicability because to scientific advancements in the field of microbial pigments over the past few decades. According to the results of the current study, the synthesis of biopigment prodigiosin can be used as a well‐thought‐out design of medicinal products made via bacterial fermentation. The wide range of anticancer activity demonstrated by prodigiosin pigment against various cell types identified it as a promising option for cancer treatment. On HepG2 cancer cells, the prodigiosin pigment derived from *Serratia marcescens* VITSD2 exhibited strong cytotoxicity. This study may contribute to the validation of the detail that prodigiosin could be used in the treatment of liver cancer. The experimental result from the study reveals that the pigment prodigiosin from *Serratia marcescens* VITSD2 could serve as an effective biological agent against cancer. Further in vivo studies are needed to unravel the mechanistic action of the pigment on cancer cells. Hence the bioactive pigments of bacterial origin could serve the mankind for treating various diseases and highlights its significance. Further research on these studies will provide promising avenues for biomedical and pharmaceutical research.

## Author Contributions

**Shah Alam:** investigation, writing – original draft. **Suraj Kumar Nag:** investigation, writing – original draft. **Jemima Naine S.:** investigation, validation, data curation, writing – review and editing. **Mohanapriya A.:** software, validation. **Sreelakshmi R. Nair:** validation, writing – review and editing. **Tamil Bharathi Palanisamy:** software, validation and in silico *analysis*. **Mohanasrinivasan V.:** supervision, writing – review and editing, validation. **Subathra Devi C.:** conceptualization, methodology, validation, formal analysis, supervision, writing – review and editing.

## Ethics Statement

The authors have nothing to report.

## Conflicts of Interest

The authors declare that the research was conducted in the absence of any commercial or financial relationships that could be construed as a potential conflict of interest.

## Data Availability

The data that support the findings of this study are openly available in NCBI‐Gen Bank at https://www.ncbi.nlm.nih.gov/nuccore/HQ197382, reference number HQ197382. The nucleotide sequence data generated and analyzed during the current study have been deposited in the GenBank database under the accession number. https://www.ncbi.nlm.nih.gov/nuccore/HQ197382.
